# FT-Raman data analyzed by multivariate and machine learning as a new methods for detection spectroscopy marker of platinum-resistant women suffering from ovarian cancer

**DOI:** 10.1038/s41598-023-48169-3

**Published:** 2023-11-26

**Authors:** Marta Kluz-Barłowska, Tomasz Kluz, Wiesław Paja, Jaromir Sarzyński, Monika Łączyńska-Madera, Adrian Odrzywolski, Paweł Król, Józef Cebulski, Joanna Depciuch

**Affiliations:** 1 Department of Pathology, Fryderyk Chopin University Hospital, F. Szopena 2, 35-055 Rzeszow, Poland; 2 Department of Gynecology, Gynecology Oncology and Obstetrics, Fryderyk Chopin University Hospital, F.Szopena 2, 35-055 Rzeszow, Poland; 3 Institute of Medical Sciences, Medical College of Rzeszow University, Kopisto 2a, 35-959 Rzeszow, Poland; 4 Institute of Computer Science, College of Natural Sciences, University of Rzeszow, Rzeszow, Poland; 5 Department of Biochemistry and Molecular Biology, Medical University of Lublin, 20-093 Lublin, Poland; 6 College of Medical Sciences, Institute of Physical Culture Studies, University of Rzeszow, 35-959 Rzeszów, Poland; 7 Institute of Physics, College of Natural Sciences, University of Rzeszow, 35959 Rzeszow, Poland; 8 Institute of Nuclear Physics Polish Academy of Sciences, 31342 Krakow, Poland

**Keywords:** Biomarkers, Oncology, Gynaecological cancer

## Abstract

The phenomenon of platinum resistance is a very serious problem in the treatment of ovarian cancer. Unfortunately, no molecular, genetic marker that could be used in assigning women suffering from ovarian cancer to the platinum-resistant or platinum-sensitive group has been discovered so far. Therefore, in this study, for the first time, we used FT-Raman spectroscopy to determine chemical differences and chemical markers presented in serum, which could be used to differentiate platinum-resistant and platinum-sensitive women. The result obtained showed that in the serum collected from platinum-resistant women, a significant increase of chemical compounds was observed in comparison with the serum collected from platinum-sensitive woman. Moreover, a decrease in the ratio between amides vibrations and shifts of peaks, respectively, corresponding to C–C/C–N stretching vibrations from proteins, amide III, amide II, C = O and CH lipids vibrations suggested that in these compounds, structural changes occurred. The Principal Component Analysis (PCA) showed that using FT-Raman range, where the above-mentioned functional groups were present, it was possible to differentiate the serum collected from both analyzed groups. Moreover, C5.0 decision tree clearly showed that Raman shifts at 1224 cm^−1^ and 2713 cm^−1^ could be used as a marker of platinum resistance. Importantly, machine learning methods showed that the accuracy, sensitivity and specificity of the FT-Raman spectroscopy were from 95 to 100%.

## Introduction

Ovarian malignancies are heterogenous neoplasms of the distinct origin, precursor lesions, clinical course, risk factors, molecular profiles, treatment and outcomes. Histologically, WHO classifies the ovarian malignancies in distinct groups based on their origin, the most frequently encountered are epithelial ovarian cancers (EOC), constituting 90% of malignant ovarian tumors^1^.

The data published by GLOBOCAN in 2020 showed that there had been 313 959 new cases and 207 252 deaths due to ovarian cancer, which made it 8th most commonly diagnosed cancer in females and was ranked 8th as far as cause of cancer deaths is concerned^2^. Interestingly, ovarian carcinoma is the leading cause of death in women diagnosed with gynecological cancers^3, 4^. Since there is no reliable screening program and the course of disease is often asymptomatic, many patients have clinically advanced cancer at the time of the diagnosis. The staging is determined by FIGO classification which is based on both surgical and histopathological assessment. In the vast majority of cases the therapy of ovarian cancer cannot be based solely on cytoreductive surgery, and systemic treatment is crucial.

Platinum-based compounds are currently in use not only for ovarian cancer, but also lung cancer, head and neck cancer, breast cancer and many others. Platinum-based compounds target cancer cells by forming adducts/crosslinks with DNA purine bases, with a preference for guanine. These crosslinks result in DNA damage that impedes proper genome replication, transcription, and triggers cell apoptosis^5^. The accumulation of platinum antitumor agents inside the cells is the necessary assurance of cytotoxicity, so decreased influx or increased efflux is responsible for platinum resistance^6^.

Nowadays, the 1^st^ line treatment in ovarian carcinoma consists of the following: either cytoreductive surgery or neoadjuvant chemotherapy, addition of bevacizumab to carboplatin and paclitaxel to the protocol, and in the case of positive response to platinum-based compounds, and the implementation of PARP inhibitors^7^. However, up to 25% of women with ovarian cancer have so-called platinum-refractory disease. Even if patients are sensitive to 1^st^ line platinum therapy, they may develop recurrence and acquire progressive resistance over time^8^. Generally, patients treated for ovarian cancer may be divided into the following categories:

- Platinum refractory—disease progressing during therapy or within 4 weeks after the last dose
- Platinum resistant—disease progressing within 6 months of platinum-based therapy
- Partially platinum sensitive—disease progressing between 6 and 12 months
- Platinum sensitive—disease progressing with an interval of more than 12 months^9, 10^.

Both platinum refractory and platinum resistant groups of patients have poorer prognosis and are perfect candidates for clinical trials. Drugs used in such cases include cisplatin (PLD) and paclitaxel, sometimes in combination with bevaciziumab as the first line chemotherapy. As the second line we usually apply topotecan, gemcitabine and doxorubicin. Cisplatin is able to crosslink with the purine bases on the DNA causing the DNA damage. Paclitaxel targets microtubules causing mitotic arrest. On the other hand bevacizumab inhibits VEGF (Vascular endothelial growth factor). Topotecan binds to the topoisomerase I—DNA complex and prevents relegation of these single strand breaks. Gemcytabine interferes with DNA synthesis and targets ribonucleotide reductase. Doxorubicyn inhibits the progression of topoisomerase II. Drugs used in such cases include topotecan, PLD, gemcitabine and paclitaxel in combination with bevacizumab. However, cisplatin is used as the first anyway. Therefore, a method is needed to determine whether a given patient is platinum-sensitive or platinum-resistant in order to increase her chance of recovery. The Raman spectroscopy may be a method. This technique provides information about chemical characterization of the sample under the study. Consequently, chemical fingerprint of the analyzed sample could be obtained^11^. Moreover, the Raman spectroscopy is a fast, non-invasive, non-expensive and non-destructive technique, which means, that in a short time results could be obtained^12^. Furthermore, a small amount of sample is needed, which causes that in a lot of medical and biological research the Raman spectroscopy is used, also in oncology research, e.g. to differentiate cancer and non-cancer tissues^13^, to show effectiveness of a treatment^14^ or to determine tumor resection margin^15^. Consequently, in this study, for the first time, chemical differences occurred in the serum collected from platinum-sensitive and platinum-resistant women suffering from ovarian cancer using the FT-Raman spectroscopy will be determined. To show significance of these differences, a statistical analysis will be performed. In order to show differentiation between the analyzed samples, a multivariate analyses will be done. Finally, to show accuracy, selectivity and sensitivity of the FT-Raman spectroscopy, a machine learning (ML) method will be used. For this purpose, four ML algorithms were applied: decision trees C5.0, Random Forest (RF), k-Nearest Neighbors (kNN) and Support Vector Machine (SVM). The first one was used to obtain information about the most significant Raman shifts, which could be used as a FT-Raman marker of platinum-resistance. In turn, random forest is an ensemble learning algorithm that combines multiple decision trees to create a more robust and accurate model. Next, SVM algorithm was used to find a hyperplane that maximizes the margin between different classes while minimizing classification errors. Finally, The KNN algorithm was used to measure the similarity between data points. For classification tasks, the class labels of the k nearest neighbors are used to predict the class of the query point.

## Results

**Figure 1:**
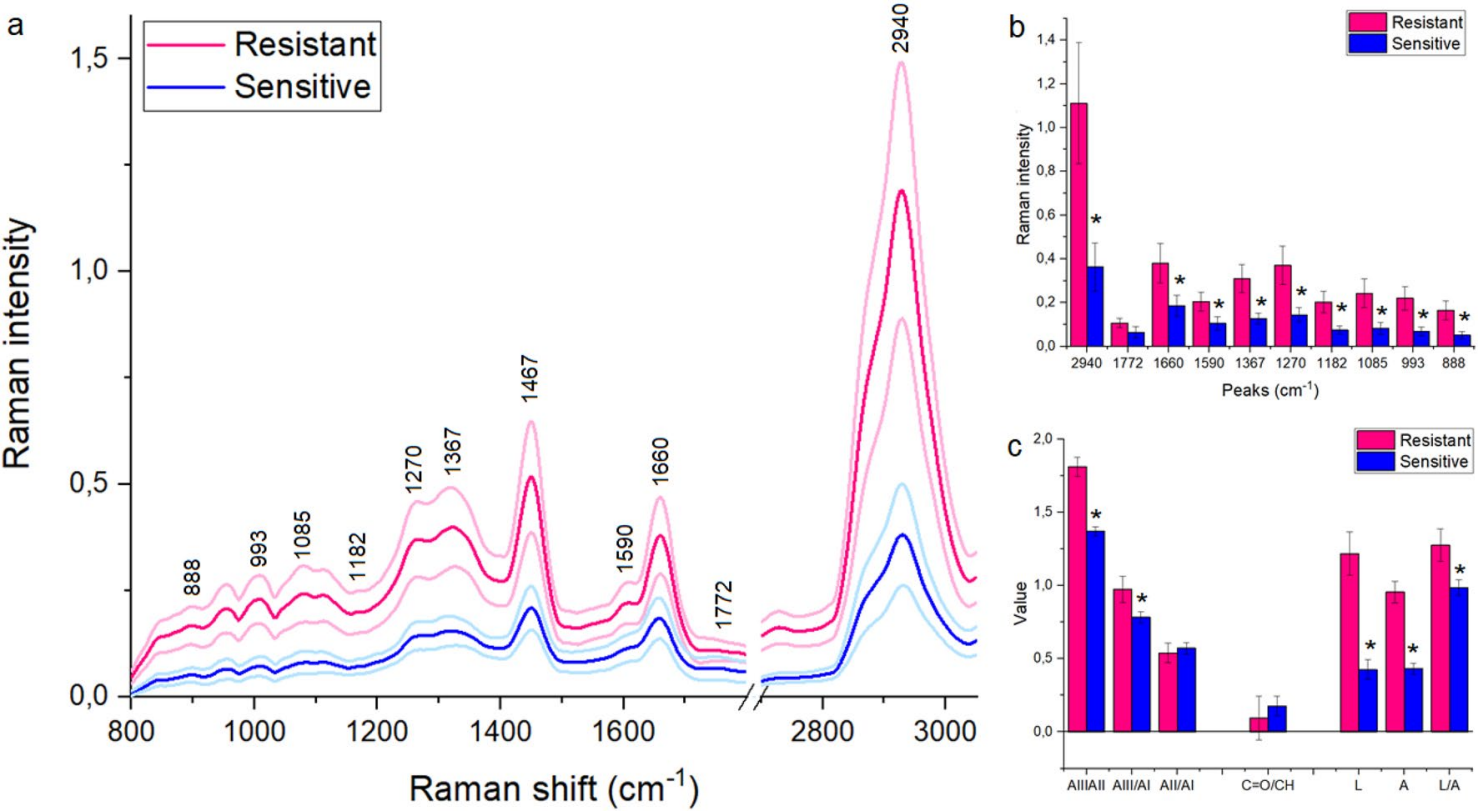
Average of FT-Raman spectra ± Standard Deviation (SD) of serum collected from platinum-sensitive (blue curve) and platinum-resistant (pink color) women suffering from ovarian cancer (**a**); average value of peaks intensities ± SD, where # mean significant differences between platinum-sensitive and platinum-resistant woman. The degree of significance was denoted as p < 0.05 (**b**); the ratio between respectively amides and lipids vibrations, as well as the sum of amides (= A), lipids (= L) and the ratio between lipids and amides (L/A) (**c**).

The FT-Raman spectroscopy was used to determine chemical differences in the serum collected from women suffering from ovarian cancer, where one part of patients were platinum-resistant women and the second part—platinum-sensitive ones. In both the FT-Raman spectra vibrations from hydroxyproline (~ 890 cm^−1^), C-H in-plane bending mode (~ 1000 cm^−1^) and C–C vibrations (~ 1090 cm^−1^) was noticed^16^. Moreover, CH_3_ bonds from lipids and proteins were visible around ~ 1370 cm^-1^^17^. Amide III, amide II and amide I vibrations were located at ~ 1280 cm^−1^, ~ 1550 cm^−1^ and ~ 1670 cm^−1^ Raman shifts^18^, while lipids C = O and C–H vibrations were visible around ~ 1770 cm^−1^ and ~ 2950 cm^−1^^19^. Moreover, the additional peak around 1467 cm^–1^ corresponding CH_2_ bonds from lipids and proteins was visible in the FT-Raman spectra of serum collected from platinum-sensitive women^17^, Fig. 1a. The detailed positions of the described peaks for each analyzed diseases were located in Table 1.

**Table 1:** Peaks analyzed in the FT-Raman spectra of platinum-sensitive and platinum-resistant patients with a band assigned and differences between positions, where “*” means the shift higher than 8 cm^−1^.

Sensitive (S) (cm^−1^)	Resistant (R) (cm^−1^)	S–R (cm^−1^)	Band assigned
888	892	− 4	Hydroxyproline
993	996	− 3	C-H in-plane bending mode
1085	1085	0	C–C
1182	1197	− 15*	C–C/C–N stretching vibrations from proteins
1270	1286	− 16*	Amide III
1367	1371	− 4	CH_3_ band from proteins and lipids
1467	1452	15*	CH_2_ from lipids and proteins
1590	1590	0	Amide II
1660	1668	− 8	Amide I
1772	1783	− 11*	C55O from lipids
2940	2964	− 24*	Cholecterol and cholesterol ester

Figure 1b showed that in the FT-Raman spectrum of serum collected from platinum-resistant women a significant increase of hydroxyproline (888 cm^−1^), C–C band (1085 cm^−1^), C–C/C-N stretching vibrations from proteins (1182 cm^−1^), three amides vibrations (1270 cm^−1^, 1590 cm^−1^, 1660 cm^−1^), CH_3_ and CH_2_ groups from lipids and proteins (1467 cm^−1^, 1367 cm^−1^, respectively), as well as CH lipids vibrations (2940 cm^−1^) were observed in comparison with the FT-Raman spectrum of serum collected from platinum-sensitive woman. Moreover, Fig. 1c showed an increase in the ratio between (i) amide III/amide II, (ii) amide III/amide II in platinum-resistant woman in comparison with the platinum-sensitive one. Moreover, higher lipids, as well as amides level was observed in the platinum-resistant women. In the same group an increase in the ratio between lipids and amides vibrations was noticed.

Table 1 showed that in the FT-Raman spectrum of serum collected from platinum-resistant woman, a significant shift of peaks corresponding to C–C/C–N stretching vibrations from proteins, amide III, CH_2_ from lipids and proteins, C = O and CH lipids vibrations toward lower Raman shifts were observed. These suggested that in these chemical compounds changes in the structure could occur.

**Figure 2:**
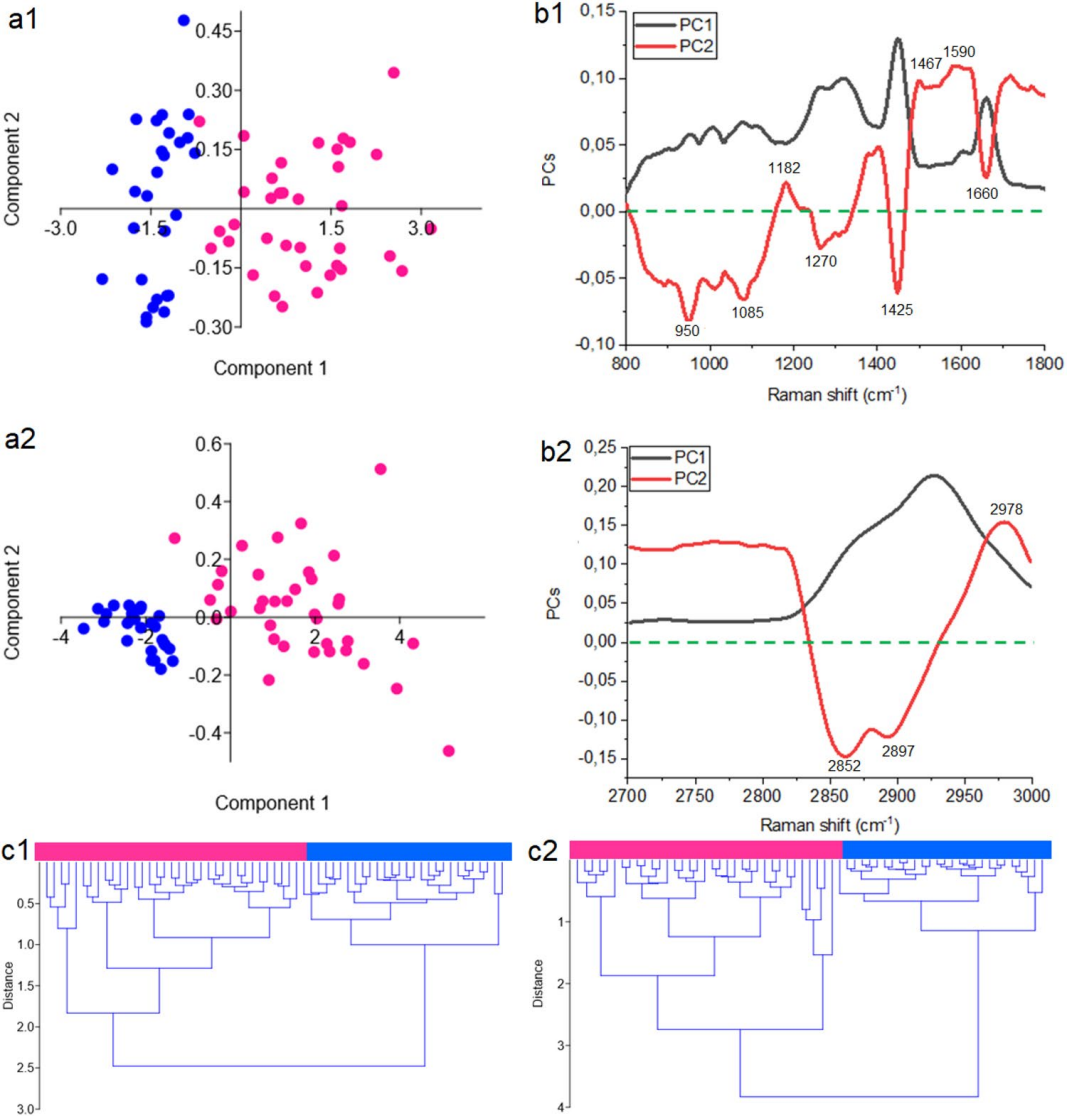
PCA analysis (**a**) with loading plots (**b**) and HCA analysis (**c**) of FT-Raman data of serum collected from platinum-sensitive (blue curve) and platinum-resistant (pink color) women suffering from ovarian cancer. The analyses were done using two spectral ranges: 800–1800 cm^−1^ range (1) and 2800–3000 cm^−1^ (2).

To show if it was possible to differentiate serum collected from platinum-resistant and platinum-sensitive women using the FT-Raman spectroscopy, the PCA analysis was performed, Fig. 2a. This analysis was done for two FT-Raman ranges: 800–1800 cm^−1^ and 2800–3000 cm^−1^. In the case of the first range, the PCA analysis showed that the samples collected from platinum-sensitive women had the negative value of PC1, and positive as well as the negative value of PC2, Fig. 2a1. Therefore, PC1 played the most important role in distuinguishing the samples collected from two analyzed groups of women. Loading plots of PC2 showed positive peaks at 1182 cm^−1^, 1467 cm^−1^ and 1590 cm^−1^, 1660 cm^−1^ Raman shifts and negative peaks at 950 cm^−1^, 1085 cm^−1^, 1270 cm^−1^ and 1425 cm^−1^ Raman shifts, Fig. 2b1. In the Raman range between 2800 cm^-1^ and 3000 cm^−1^, the values of PC1 and PC2 for the samples collected from platinum-resistant and platinum-sensitive women were the same as for ranges 800–1800 cm^−1^, Fig. 2a2. In the loading plots negative peaks at 2852 cm^−1^ and 2897 cm^−1^ and one negative peak at 2978 cm^−1^ were noticed, Fig. 2b2.

Furthermore, HCA analysis showed creation of two similarity groups, where in the first group all the samples collected from platinum-sensitive women were placed, and in the second one—all the samples from other analyzed group. It was visible for both analyzed FT-Raman regions, Fig. 2c1,c2. The results suggested that changes in the proteins and lipids could play an important role in platinum resistance phenomenon.

To sum up, the FT-Raman results showed significant differences in the chemical compositions of serum collected from platinum-resistant and platinum-sensitive woman. Consequently, machine learning methods were used to show accuracy, selectivity and specificity of this technique. The results of the analysis of four created datasets using ML algorithms were shown in Table 2. The calculated values of parameters showed that accuracy was from 95 to 100%, sensitivity—from 89 to 100% and specificity from 97 to 100%.

**Table 2:** FT-Raman mean values of the quality assessment parameters of the four obtained learning algorithms, using 4 datasets.

Dataset	No. of features	Algorithm	Accuracy	Sensitivity	Specificity	Precision	f1	mcc
800–1800 cm^−1^	260	C5.0	0.95	0.89	1.00	1.00	0.91	0.94
		RF	0.98	1.00	0.97	0.96	0.97	0.98
		kNN (k = 1)	0.98	1.00	0.97	0.96	0.97	0.98
		SVM	0.98	1.00	0.97	0.96	0.97	0.98
800–1800 selected wavenumbers	176 selected	C5.0	0.95	0.89	1.00	1.00	0.91	0.94
		RF	0.98	1.00	0.97	0.96	0.97	0.98
		kNN (k = 1)	0.98	1.00	0.97	0.96	0.97	0.98
		SVM	0.98	1.00	0.97	0.96	0.97	0.98
2800–3000 cm^−1^	79	C5.0	0.98	0.96	1.00	1.00	0.97	0.98
		RF	0.98	1.00	0.97	0.96	0.97	0.98
		kNN (k = 1)	0.98	1.00	0.97	0.96	0.97	0.98
		SVM	1.00	1.00	1.00	1.00	1.00	1.00
2800–3000 selected wavenumbers	79 selected	C5.0	0.98	0.96	1.00	1.00	0.97	0.98
		RF	0.98	1.00	0.97	0.96	0.97	0.98
		kNN (k = 1)	0.98	1.00	0.97	0.96	0.97	0.98
		SVM	1.00	1.00	1.00	1.00	1.00	1.00

**Figure 3:**
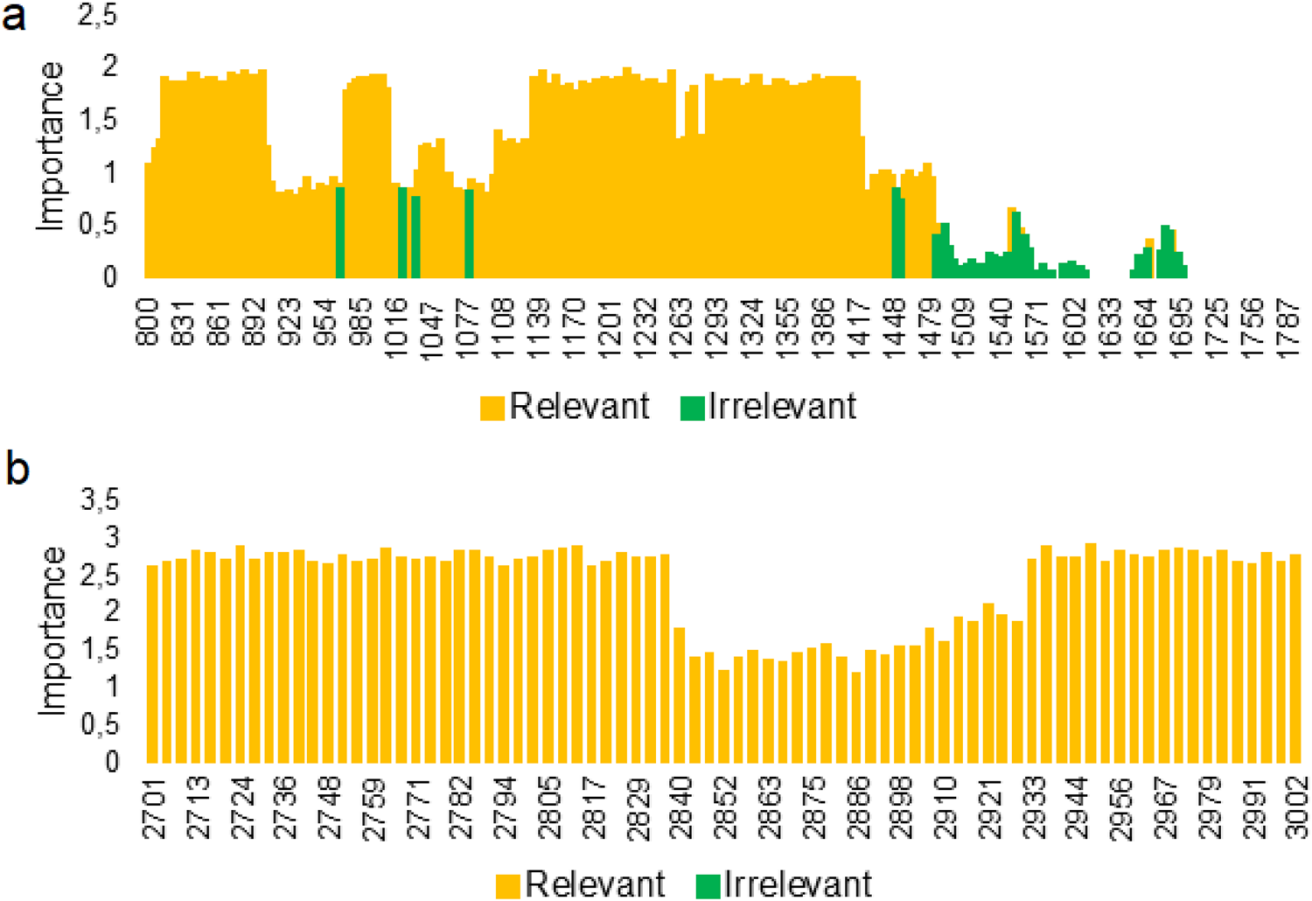
Significant ranges of Raman shifts in the differentiation serum collected from platinum-sensitive and platinum-resistant woman obtained using machine learning algorithms. Significant ranges were calculated for two Raman ranges: 800–1800 cm^−1^ (**a**) and 2700–3000 cm^−1^ (**b**).

The analysis of the difference in the spectrum of Raman intensity values (Fig. 3, Table 3) for the categories of positive and negative cases shows that about 6 Raman shifts ranges can be identified, which clearly distinguishes the serum collected from platinum-sensitive and platinum-resistant women.

**Table 3:** Identified significant ranges of wavenumbers.

Raman 800–1800 cm^−1^	800–962 cm^−1^
	969–1016 cm^−1^
	1035–1074 cm^−1^
	1081–1444 cm^−1^
	1455–1479 cm^−1^
Raman 2800–3000 cm^−1^	2700–3000 cm^−1^

For the 800–1800 cm^−1^ analyzed FT-Raman range, a strong difference in absorption was seen at the 800–926 cm^−1^, 969–1016 cm^−1^, 1035–1074 cm^−1^, 1081–1441 cm^−1^, 1455–1479 cm^−1^ and 2700–3000 cm^−1^ ranges, Table 3. Consequently, these ranges could be used as strong markers that differentiate platinum-sensitive and platinum-resistant women.

**Figure 4:**
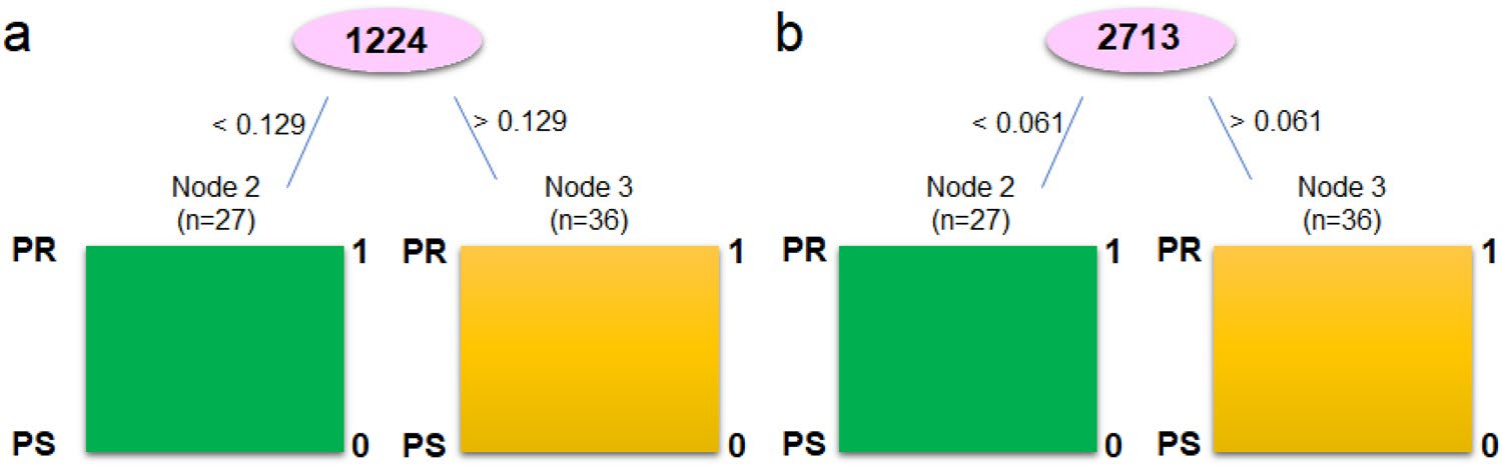
Decision tree for FT-Raman ranges 800–1800 cm^−1^ (**a**) and 2700–3000 cm^−1^ (**b**).

Also, the C5.0 decision tree learning model generated from these data (Fig. 4) indicates that 1224 cm^−1^ was the most important Raman shift, and was a value that unambiguously distinguishes the two categories of patients. Similarly, for the 2800–3000 cm^−1^ range, a another FT-Raman range under the study, could be used as a marker that differentiates platinum-sensitive and platinum-resistant women.

## Discussion

The survival rates of the ovarian cancer are poor. It is caused by advanced disease stages and disease recurrence, which results of platinum chemotherapy resistance^20^. Until now, ovarian cancer has been treated by paclitaxel/carboplatin combination chemotherapy, and unfortunately the majority of patients became platinum-resistant with subsequent relapses of the disease^8^. Moreover, platinum resistance can be detected only after 6 months, which is associated with a decrease in patients' chances of recovery^21^. Therefore, it is needed to find platinum-resistance biomarkers, which will show if patient should be treated by platinum compounds or not since in histopathological examination we cannot observe any indicators which will differentiate these two groups. Moreover, also the mechanism of platinum-resistant in ovarian cancer is still not well known, which causes additional problems in chemotherapy of this kind of cancer. Ottevanger et al. showed that the most probable mechanism of platinum resistant is the fact that during chemotherapy it remains, the cancer stem cells remain at rest, consequently drugs have no effect on cancer stem cells^22^. Nowadays, the most likely marker of platinum resistance is bone morphologenetic protein 2 (BMP2), which is upregulated in ovarian cancer cells, and which is correlated with poor prognosis^23, 24^. However, although the evidence, that these proteins could be biomarkers of platinum resistance is compelling, prognostic testing or development of targeted treatments are still not accessible. Moreover, only by means of expensive methods, the level of BMP2 in platinum-resistant and platinum-sensitive patients can be investigated. Therefore, in this study, we wanted to investigate if it is possible to find another platinum-resistance marker, which could be found fast and, which will be inexpensive. For this purpose, the FT-Raman spectroscopy in combination with machine learning and multivariate analyses were used. Indeed, the results obtained showed that the greatest differences between platinum-sensitive and platinum-resistant patients occurred in the region of vibration of protein functional groups (Fig. 1a,b), which reflected the proposal of BMP2 protein as a marker of platinum resistance^23, 24^. Furthermore, in platinum-resistant women suffering from ovarian cancer, higher expression of low density lipoprotein receptor (LDLR) was visible^25^, which also showed that the FT-Raman marker region obtained could have a potential application. This is even more convincing as the result of C5.0 decision tree method indicated the amide II region as a potential spectroscopic marker region, Table 3. Moreover, in platinum-resistant women, higher amount of amides, as well as changes in the amides balance was observed (Fig. 1c), which suggested, that changes in the proteins expression and structure occurred^25^. However, analyses of the FT-Raman spectra also showed that C-H lipids functional groups could be used as a platinum-resistant marker (Figs. 2a2, 4b Table 3). Indeed, in chemotherapy, where platinum drugs were used, the fatty acid binding protein 4 (FABP4) plays a very important role as itis responsible for promotion the uptake of long chain fatty acids into cells^26, 27^. Consequently, in platinum-sensitive woman a lower amount of free fatty acid should be noted. Our results showed that in platinum-resistant women higher amount of lipids functional groups, as well as global lipids fraction and changes in the lipids balance were observed, Fig. 1. Moreover, PCA analysis of lipids FT-Raman region clearly showed that using this FT-Raman range it was possible to differentiate platinum-resistant and platinum-sensitive women with ovarian cancer. Importantly, in this study we used baseline correction and normalization of all obtained spectra using the same methods. It means that we minimalized the influence of sample thickness and homogeneity changes on the results obtained, especially quantitative ones. Moreover, we used the same volume of samples for measurements. Taking into account results obtained by other Authors, which used molecular biology methods^23–27^ and these which we showed, the correlation between the data obtained by different techniques was visible and, what is the most important, has a sense with medical data about platinum-resistant mechanism.

Summarizing, we showed, that using the FT-Raman spectroscopy, functional groups distinguishing samples obtained for two analyzed groups of women suffering from ovarian cancer could be found. These functional groups build protein and lipid structures. In the case of the protein structures, the results are consistent with protein molecular platinum-resistant markers^23, 28^. The indication of lipids as markers of platinum resistance is a novelty, however, the correlation with molecular studies also exists^26^. Additionally, taking into account the fact that PCA, HCA and machine learning methods confirm that lipids may have an impact on platinum resistance, we believe that the result is valid and noteworthy. However, further research in this direction should be conducted.

## Conclusions

In the presented study, the FT-Raman spectroscopy was used to determine chemical differences in serum collected from platinum-resistant and platinum-sensitive women. Moreover, using multivariate and machine learning methods the spectroscopy data was analyzed to show specific chemical features which could be used to differentiate both studied groups of serum. The results obtained clearly showed that quantitative and qualitative differences between serum collected from both groups of women suffering from ovarian cancer existed. In the FT-Raman spectrum of serum collected from platinum-resistant women significant increase of functional groups building hydroxyproline, three amides vibrations, CH_3_ and CH_2_ groups from lipids and proteins, as well as CH lipids vibrations was observed in comparison with the FT-Raman spectrum of serum collected from platinum-sensitive woman. Furthermore, structural differences were visible as changes in the value ratio between, respectively, amides vibrations and shift of peak at 1182 cm^−1^, 1270 cm^−1^, 1590 cm^−1^, 1772 cm^−1^ and 2940 cm^−1^. Using these differences, it is possible to differentiate serum collected from both groups of women, which was presented in PCA and C5.0 decision tree methods. Importantly, calculated values of parameters showed that accuracy, sensitivity and specificity were around 95%. Consequently, the results showed a possibility of using the FT-Raman spectroscopy to determine if women were platinum resistant or not. Of course, larger studies with more cases from each category have to be required to confirm these results. Moreover, these are the first studies of this type.

## Materials and methods

### Participants

All the patients who participated in this study signed informed consent. Moreover, the study was approved by the Bioethics Committee of the Regional Medical Chamber in Rzeszow–24 November 2016 (Resolution No. 90/B/2016). Furthermore, all research was performed in accordance with relevant guidelines/regulations, and from all participants (patients) the consent was obtained.

The study group of patients consisted of 21 women with histopathological diagnosis of high-grade ovarian adenocarcinoma who were treated at the Fryderyk Chopin University Hospital in Rzeszow between 2017 and 2020. Among them, 12 were platinum-resistant and 9 were platinum-sensitive. All the samples were collected during the primary surgery before any adjuvant therapy (i.e. chemotherapy, immunotherapy, hormonal therapy and radiotherapy. All of the patients were ranked as stage III according to FIGO (International Federation of Gynecology and Obstetrics) classification and the histopathological type was endometrioid Adenocarcinoma. None of the patients had any type of malignancy ever diagnosed in medical history. The average age of patients was 52,3. In the study group 63% of patients were suffering from arterial hypertension and 27% from obesity. As far as genetic studies are concerned, based on next-generation sequencing (NGS) no mutation, neither in BRCA1 and BRCA 2 gene, nor homologous recombination deficiency (HRD) were found.

### Methods

All obtained blood samples were centrifuged for 15 min at 3000 rpm to obtain pure serum. Next, the serum samples were stored at − 80 °C. The samples were dispersed just before taking measurements on the FT-Raman spectrometer.

### FT-Raman measurements

FT-Raman spectra were recorded using a Nicolet NXR 9650 FT-Raman spectrometer (Thermo Fisher Scientific, USA). In this spectrometer an Nd:YAG laser (1064 nm) and a germanium detector was used. The measurements were performed in the range from 150 to 3.700 cm^−1^ with a laser power of 1 W. Unfocused laser beam was used with a diameter of approximately 100 μm and a spectral resolution of 8 cm^−1^. Raman spectra were processed by the Omnic/Thermo Scientific software based on 64 scans. The spectra were normalized using vector normalization in OPUS 7.0 software (Bruker Optik GmbH, Ettlingen, Germany). Moreover, each spectrum was smoothed using 21 points by Savitzky–Golay algorithm.

### Analyses of FT-Raman spectra

To determine significant differences in the Raman intensity of peaks presented in the FT-Raman spectra of serum collected from platinum-resistant and platinum-sensitive women, one-way ANOVA with Tukey’a post hoc test was done using Past 4.0. software. Furthermore, to show differences in the ratio between amides and lipids vibrations, respectively, the ratio between Raman intensity of peaks originating from amide III, amide II, amide I, as well as C = O and C-H lipids vibrations were used. Finally, to determine global amount of amides (= A) and lipids (= L), the sum of the intensities of peaks at 1270 cm^−1^, 1590 cm^−1^ and 1660 cm^−1^ (amides) and the sum of intensities of peak at 1772 cm^−1^ and 2940 cm^−1^ (lipids) was calculated. Furthermore, using results of these sums, the ratio between lipids and amides (L/A) was shown. Significance was defined as less than a 0.05 p-value. Moreover, to obtain information about differentiation as well as similarity between the samples collected from platinum-resistant and platinum-sensitive women, the spectroscopic data was analyzed using PCA and HCA with Euclidean distance and paired group (UPGMA) algorithm analyses. For this purpose, two different FT-Raman range were analyzed: 800–1800 cm^−1^, where 260 points was taken and between 2700 cm^−1^ and 3000 cm^−1^ (52 points). Both analyses were performed using in Past software (developed by Oyvind Hammer). Next, in order to apply ML algorithms, the data obtained from FT-Raman spectroscopy experiments were transformed into the form of a so-called decision system. Such the system takes the form of a decision table wherethe columns denote features describing the object (in this case, Raman shifts), while the rows are specific learning objects (in this case, disease cases). The last column in the table is a decision column containing the value of the category class assigned to each learning object (in this case, the platinum-sensitive or platinum-resistant class). This form of data was the input for the ML algorithms. To sum up, the columns represent the individual: 260 Raman shifts for the 800–1800 cm^−1^ range and 79 Raman shifts for the 2700–3000 cm^−1^ range. The rows represents the Raman intensity values of these Raman shift for specific patients, the last column is the patient category: resistant (positive) and non-resistant (negative) to cisplatin. Four ML algorithms were used for the analysis: decision trees C5.0, Random Forest (RF), k-Nearest Neighbors (kNN) and Support Vector Machine (SVM). C5.0 is a popular decision tree algorithm for data mining and machine learning. It is primarily used for classification tasks, where the goal is to assign a label or a category to a set of input features. C5.0 works by creating a decision tree that recursively splits the data into subsets based on the most informative attributes, ultimately leading to a classification for each instance. In turn, random forest is an ensemble learning algorithm that combines multiple decision trees to create a more robust and accurate model. It was introduced as an improvement over individual decision trees, which can suffer from overfitting and instability. Multiple decision trees are built independently using the bootstrapped datasets and the randomly selected features. Then each tree "votes" for a class, and the class with the most votes becomes the predicted class. The primary goal of SVM algorithm is to find a hyperplane that maximizes the margin between different classes while minimizing classification errors. The margin is the perpendicular distance between the hyperplane and the nearest data point of each class. Support vectors are the data points closest to the hyperplane, and they have an influence on the position and orientation of the hyperplane. These support vectors are used to calculate the margin and determine the optimal hyperplane. The KNN algorithm makes predictions based on the similarity between a query point and its k nearest neighbors in the training dataset. KNN relies on a distance metric (e.g., Euclidean distance, Manhattan distance, or others) to measure the similarity between data points. For classification tasks, the class labels of the k nearest neighbors are used to predict the class of the query point. These models were tested on test cases using a leave-one-out cross validation (LOOCV) approach. Additionally, to determine the most significant differences (specific Raman shifts), the process of relevant feature selection will be carried out, known in machine learning, For this purpose, an algorithm will be used to assess the relevance of features using a random forest. Feature selection using a Random Forest algorithm is a technique that leverages the importance scores assigned to features by a Random Forest model to select the most relevant features for a given task. Random Forests are well-suited for this purpose because they can assess feature importance based on the impact of individual features on the model's performance. Common measures of feature importance in Random Forest include Gini impurity, mean decrease in impurity, or mean decrease in accuracy. The features with higher scores are considered more important, while those with lower scores are less important. In addition, the Boruta algorithm will be used to distinguish between relevant and irrelevant features. Boruta is based on the concept of "shadow features" that are essentially duplicates of original features from dataset. The shadow features make it possible to delineate relevant features from irrelevant features in such a way that all original features having a significance higher than the shadow features are considered essential features.

## Data Availability

The data supporting this study's findings are available from the corresponding author upon reasonable request.
